# Outcomes of Sling Removal in Women with Chronic Voiding Dysfunction: A Retrospective Observational Study

**DOI:** 10.3390/healthcare13131517

**Published:** 2025-06-25

**Authors:** Clarissa Costa, Marta Barba, Desirèe De Vicari, Alice Cola, Matteo Frigerio

**Affiliations:** 1 Department of Gynecology and Obstetrics, University of Milano-Bicocca, 20126 Monza, Italy; c.costa14@campus.unimib.it (C.C.); d.devicari@campus.unimib.it (D.D.V.); 2 Fondazione IRCCS San Gerardo dei Tintori, 20900 Monza, Italy; m.barba8792@gmail.com (M.B.); alice.cola1@gmail.com (A.C.)

**Keywords:** voiding dysfunction, chronic urinary retention, synthetic suburethral slings, urodynamics, surgery, postoperative complications

## Abstract

Stress urinary incontinence (SUI) is a common condition that can significantly impair a woman’s quality of life. While initial management includes conservative treatments, surgical options are recommended in refractory cases. Suburethral slings are currently one of the most widely recommended surgical treatments due to their high long-term efficacy. However, complications such as postoperative urinary retention can occur and may lead to chronic voiding dysfunction when not promptly diagnosed and managed. Background/Objectives: The aim of this study was to evaluate the long-term voiding function in women undergoing delayed sling removal or incision for neglected chronic voiding dysfunction. Methods: This retrospective study examined women with chronic voiding dysfunction occurring at least one year after suburethral sling placement for SUI. Preoperative evaluation included comprehensive history, physical examination, urethral ultrasound, uroflowmetry, and urodynamic studies. Surgical interventions consisted of partial or complete sling removal or sling incision (unilateral or bilateral). Postoperative follow-up was conducted at one month and then at 12 months, including clinical examination, ultrasound, and symptom assessment. Results: Sixteen patients were included in the study, all of whom presented with urodynamic confirmation of voiding dysfunction. Following surgical intervention, a significant improvement was observed in voiding symptoms and overall symptoms (*p* < 0.01). Notably, only one patient experienced persistent voiding difficulties, although complete symptom remission was achieved following sacral neuromodulation. Conclusions: Sling removal or incision has proven to be an effective approach in resolving the majority of cases of neglected chronic voiding dysfunction. These findings suggest that, when appropriately performed, surgical intervention can substantially alleviate symptoms and improve patient well-being, providing an effective therapeutic option for what is often a debilitating condition.

## 1. Introduction

Stress urinary incontinence (SUI) is a common condition defined by the involuntary loss of urine triggered by activities that increase intra-abdominal pressure, such as sneezing, coughing, or laughing [1]. It affects approximately 5–20% of women and is the most prevalent type of urinary incontinence, accounting for 60% of all cases [2]. This condition can have a significant negative impact on an individual’s social, professional, domestic, and emotional well-being.

Initial treatment options primarily include conservative measures, such as weight loss, pelvic floor muscle exercises, electrical and magnetic stimulation, local hormone therapy, and vaginal laser treatments. Surgical intervention is considered when conservative methods fail [3]. Over the years, various surgical approaches have been proposed, including bladder neck suspension, urethral bulking agents injection, anterior vaginal wall repair, autologous slings, stem cell injections, and the use of mesh tapes. Currently, suburethral slings are recognized as the first-line surgical treatment due to their high long-term efficacy in resolving stress urinary incontinence symptoms [4]. First introduced in 1995 as retropubic tapes, these slings have shown promising results, but they are also associated with certain complications, particularly visceral injuries like bladder perforation [5]. In 2001, the transobturator approach was developed to minimize the risks of visceral injury related to the blind insertion of needles in the retropubic space. This technique demonstrated high efficacy and a reduced risk of visceral perforation, though it introduced new risks, such as vascular injuries and damage to the obturator nerve, which can lead to thigh pain and neurological deficits [6]. Single-incision slings (SISs) were later introduced in 2006. These slings feature a shorter tape length, limited internal dissection, and no complete passage of introducers, thereby reducing complications like major bleeding, infection, visceral injury, and neurological pain. SISs are also associated with shorter recovery times, a minimal learning curve, and excellent long-term safety and efficacy profiles [7].

Urinary retention is a frequent early complication following surgery for stress urinary incontinence. Its occurrence ranges from 2.5% to 19.5% for retropubic slings and from 1.5% to 8.6% for transobturator slings [8,9,10]. Initial treatment typically involves methods to assist bladder emptying, such as the use of an indwelling catheter or clean intermittent self-catheterization. Most patients experience temporary voiding issues that resolve on their own within 48 h to 21 days. However, between 0.3% and 4.5% of patients who undergo suburethral sling surgery may continue to experience urinary retention for more than four weeks, and these cases often necessitate surgical intervention to remove the mesh [9,11].

Bladder outlet obstruction (BOO) is suspected when patients present persistent urinary retention (lasting more than 4 weeks) or clear symptoms of incomplete bladder emptying, weak urinary stream, and straining to urinate. However, many patients may have subtler symptoms, such as urgency, frequency, and nocturia, which can make diagnosing BOO challenging. Diagnosis should be based on the patient’s history, physical examination, imaging of the lower urinary tract, and urodynamic pressure–flow studies [12].

To alleviate symptoms and prevent further bladder dysfunction, surgical intervention is recommended for postoperative BOO. Surgical options for treating BOO following sling surgery include sling incision, sling lysis, partial removal, and extensive vaginal or retropubic urethrolysis, which involves removing the sling and breaking up the fibrous tissue around the urethra and bladder neck [13]. When BOO is diagnosed long after sling surgery, urethrolysis combined with mesh transection is recommended, with success rates ranging from 70% to 85%, and a recurrence of stress urinary incontinence in about 19% of cases [14]. If a second urethrolysis is required, the resolution rate increases to about 92%, with a recurrence of incontinence similar to the first procedure [15].

In this study, we aimed to assess the urological outcomes in women who underwent sling removal due to chronic voiding dysfunctions that developed after the initial placement of synthetic suburethral slings. We specifically focused on evaluating the long-term effects of sling-related complications such as bladder outlet obstruction and persistent urinary retention, while also examining how delayed intervention influenced symptom resolution and overall patient well-being.

## 2. Materials and Methods

We conducted a retrospective observational cohort study at San Gerardo Hospital, University of Milano Bicocca, Monza, Italy, in accordance with the STROBE guidelines for observational research (Supplementary Materials). We retrospectively reviewed our institutional surgical database to identify cases of sling removal performed between January 2000 and December 2024. From this cohort, we selected patients who had been referred to our department for the assessment of chronic voiding dysfunction that developed at least one year after the implantation of a synthetic suburethral sling for the treatment of stress urinary incontinence, and who subsequently underwent sling removal.

Preoperative evaluation included a detailed clinical history, pelvic examination, and pelvic and urethral ultrasound. All patients underwent urodynamic testing prior to surgery, including uroflowmetry and pressure–flow studies, conducted by a trained urogynecologist to assess urinary retention and stress urinary incontinence. All procedures and definitions adhered to the Good Urodynamic Practice Guidelines established by the International Continence Society.

Patients were eligible for inclusion if they met the following criteria: presence of chronic voiding dysfunction, defined as persistent voiding symptoms (e.g., weak stream, straining, incomplete emptying) lasting more than 4 weeks and occurring at least 12 months after sling implantation, or urodynamic evidence of bladder outlet obstruction; and availability of complete preoperative and postoperative clinical data, including symptom assessment and a minimum follow-up of 12 months.

Patients were excluded from the study based on the following criteria: presence of neurogenic bladder disorders, history of prior anti-incontinence surgeries other than the initial midurethral sling procedure, concurrent pelvic organ prolapse greater than stage I at the time of sling removal surgery, previous radical pelvic surgeries or pelvic irradiation, incomplete medical records, or insufficient follow-up data post-sling removal surgery. These criteria were established to ensure a homogeneous study population and to minimize confounding variables that could affect the assessment of surgical outcomes following sling removal.

Depending on the clinical presentation and the surgeon’s expertise, patients underwent either single or bilateral sling incision, partial sling removal, or complete sling excision. The choice of procedure was individualized based on the severity of symptoms and intraoperative findings. Severity of symptoms was assessed based on frequency and intensity of voiding difficulties, postvoid residual volume, presence of irritative symptoms, and impact on daily life as reported by the patient. A postvoid residual volume >100 mL was considered abnormal, consistent with current urological guidelines and the International Continence Society definition.

Postoperative evaluations were conducted at 1 month and at 12 months, and included a review of urogenital symptoms, voiding diaries, physical examination, and pelvic ultrasound. Uroflowmetry and pressure–flow studies were not routinely performed in the absence of urological symptoms during follow-up.

All statistical analyses were performed using SPSS software, version 29 (IBM Corp., Armonk, NY, USA). Categorical variables were presented as absolute and relative frequencies, while continuous variables were expressed as mean ± standard deviation. Comparisons of non-continuous variables were performed using Fisher’s exact test. A *p*-value of <0.01 was considered statistically significant.

## 3. Results

Our surgical database identified 43 patients who underwent sling removal. Among these, 16 women underwent the procedure specifically for chronic urinary retention.

The mean age at the time of sling removal surgery was 64.5 ± 14.2 years. The majority of participants were menopausal, with 13 women (81.3%) reporting this status, and the average parity was 2.2 ± 0.6. A significant portion of the population (10 women, 62.5%) had a history of prior surgeries. Of these, the majority (eight women, 80%) had undergone vaginal surgery, while a smaller subset (two women, 20%) had other types of prior surgeries. No comorbidities potentially affecting the primary urinary symptoms were identified.

Sling procedures were carried out between 2000 and 2017; in eight cases, the intervention was performed at other institutions, while the remaining eight were operated on at our center. The sling types used for the treatment of stress urinary incontinence (SUI) varied. The most frequent sling type was the transobturator tape (TOT), used in 6 out of 16 patients (37.5%). The TVT (Tension-free Vaginal Tape) procedure was used in five cases (31.25%), and the SIS (Suburethral Intravaginal Sling) procedure was employed in five cases (31.25%).

The preoperative uroflowmetry parameters provided insights into the participants’ urinary function prior to surgery. The maximum urine flow rate was 12.9 ± 6.4 mL/s, and the flow time averaged 40.6 ± 21.3 s, suggesting a relatively prolonged time for urination in some patients. Preoperative urodynamic evaluation showed that six women (37.5%) presented with detrusor hyperactivity. The mean PVR on urodynamics was 162.2 ± 14.4 mL, while a positive PVR was observed in eight women (50%). No cases of USUI were observed in this cohort. Preoperative uroflowmetry and urodynamics results are shown in Table 1.

Urethral ultrasound was conducted to assess the position of the sling relative to the bladder neck and the urethral lumen. Six patients (37.5%) had the sling positioned in the middle third of the urethra, five patients (31.25%) in the distal third, and five patients (31.25%) in the proximal third. The average distance between the sling and the urethral lumen was 2.8 ± 1.1 mm.

The sling removal procedures were carried out between 2011 and 2024, with a mean time to reoperation of 12 ± 6.2 months following the original sling placement.

Surgical procedures varied among the patients: eight women underwent partial sling removal, while five women had complete sling removal. Smaller subsets of the population underwent sling incision, with one woman undergoing unilateral sling incision and two women undergoing bilateral sling incision.

Symptoms were evaluated before surgery and at the 12-month postoperative follow-up. A detailed analysis of these symptoms is presented in Table 2.

There was an overall improvement in all symptoms, except for stress urinary incontinence, which showed a slight increase after sling removal; however, this change did not reach statistical significance. Voiding difficulties decreased from 100% to 6.75% (*p*-value < 0.001), and 7 out of 16 patients became completely asymptomatic (*p*-value < 0.007).

Patients who remained symptomatic for UUI, recurrent UTIs, and SUI after sling removal were successfully treated with pelvic floor rehabilitation and/or medical therapy. Only one patient continued to experience voiding dysfunction despite conservative treatment and was subsequently referred for sacral neuromodulation, with significant symptom improvement.

## 4. Discussion

This retrospective observational study aimed to evaluate the urological outcomes in women who underwent delayed sling removal due to chronic voiding dysfunctions after synthetic suburethral sling procedures for stress urinary incontinence. The findings underscore the complexities associated with sling-related complications and highlight the impact of delayed surgical intervention on symptom resolution.

Population characteristics are in line with the typical demographic profile of women undergoing sling procedures for SUI, as age, menopausal status, and previous pelvic surgeries are important factors influencing the decision to pursue surgical interventions for incontinence. The variety in sling types used (TOT, TVT, and SIS) reflects the evolution of surgical techniques and the ongoing debate regarding the optimal approach for treating SUI. In our cohort, all three main types of midurethral slings were represented with a relatively balanced distribution. Although the study was not powered to detect differences in outcomes based on sling type, no clear association was observed between the type of sling and the severity or duration of voiding dysfunction. This aligns with previous literature suggesting that while each sling type carries distinct anatomical and technical considerations, the development of long-term complications such as bladder outlet obstruction may be influenced more by patient-specific factors, surgical technique, and tape positioning than by the sling design itself.

Urodynamic studies and uroflowmetry demonstrated a weak urinary stream in all patients, with a mean detrusor pressure at Maximum Flow (Pdet) of 25.1 cmH_2_O and a mean maximum flow rate (Qmax) of 12.2 mL/s. These values are consistent with the criteria commonly reported in the literature for diagnosing urodynamic urethral obstruction, typically defined as a Pdet greater than 20 cmH_2_O combined with a Qmax less than 15 mL/s [16]. Despite these obstructive patterns, both the Bladder Contractility Index (BCI) and Maximum Cystometric Capacity (MCC) remained within normal ranges, suggesting that detrusor contractility was largely preserved, and bladder capacity was not a limiting factor. The presence of detrusor overactivity in some patients may reflect a compensatory response of the bladder attempting to overcome the urethral obstruction. Taken together, these findings indicate that although patients exhibited signs of obstruction, bladder decompensation had not yet occurred, highlighting the importance of timely surgical intervention before irreversible functional deterioration sets in.

Ultrasound findings regarding the positioning of the sling relative to the urethra can offer valuable insight into the potential mechanisms of postoperative dysfunction, although studies report varying results. Kociszewski et al. found that urge symptoms and voiding difficulties were associated with a short distance (<3 mm) between the tape and the urethral lumen [17]. These findings align with our results, where the average distance between the sling and the urethral lumen was 2.8 mm. In a subsequent study, Kociszewski et al. observed that the most favorable outcomes were seen when the tape was positioned between 40% and 70% of the urethral length [18]. Similarly, Spelzini et al. suggested that while perfect placement of the sling at the mid-urethra may not be strictly necessary for success, dislocation of more than 10% from the mid-urethra could increase the risk of complications [19]. On the other hand, some studies have found no significant correlation between ultrasonographic findings and postoperative urinary sequelae [20]. In line with these reports, our results did not show a significant difference in sling placement relative to total urethral length. However, due to the small sample size in our study, further research is needed to fully clarify these observations.

Sling removal was performed in a variety of ways, including partial removal, complete removal, and unilateral or bilateral mesh transection. The choice of procedure was influenced by the severity of symptoms and intraoperative findings. The majority of patients underwent partial sling removal, which suggests that a more conservative approach was often preferred. The literature suggests that outcomes in cases of bladder outlet obstruction tend to improve regardless of the extent of mesh removal, and even mesh transection alone may be effective. Although adverse events are uncommon following both partial and complete mesh removal, total mesh excision carries a higher risk of complications such as hematomas, abscesses, port site issues, and a greater likelihood of incontinence recurrence. For this reason, complete removal should be considered primarily in cases where sling implantation was recent, and the dissection plane remains easily accessible [21,22,23].

From a symptom perspective, sling insertion led to a marked increase in voiding difficulties and, although without reaching statistical significance, the onset of irritative symptoms, such as urinary urgency, urge urinary incontinence, and recurrent urinary tract infections. However, these symptoms were largely resolved following sling removal, as also reported by several studies [24]. Sling removal also resulted in a partial recurrence of stress urinary incontinence symptoms without statistical significance. This outcome is consistent with previous studies reporting that SUI recurrence can be a consequence following sling removal or revision procedures. Nevertheless, the absence of a complete recurrence suggests that, in certain patients, even after sling incision, the remaining portion of the sling and the surrounding fibrotic tissue can still provide sufficient support to prevent SUI [23]. The persistence of urinary symptoms in some patients after sling removal underscores the need for ongoing treatment, including pelvic floor rehabilitation, medical therapy, and potentially more invasive interventions such as sacral neuromodulation, to optimize long-term outcomes. This emphasizes the importance of individualized treatment strategies, as some patients may require additional interventions beyond sling removal to achieve satisfactory results.

The potential role of postoperative vaginal estrogen therapy in improving outcomes after sling surgery deserves consideration. In postmenopausal women, local estrogen treatment has been shown to enhance the trophism and elasticity of the vaginal mucosa, reduce inflammation, and support tissue remodeling. These effects may contribute to a lower risk of mesh-related complications such as erosion, fibrosis, and tissue atrophy, which are known to predispose to voiding dysfunction. Although estrogen therapy was not systematically evaluated in our study, its use could represent a valuable adjunctive strategy, particularly in selected patients with hypoestrogenic status. Future prospective studies should investigate whether postoperative vaginal estrogenization may reduce the incidence or severity of late-onset voiding disorders following sling procedures [25].

This study has several limitations that should be considered. The sample size is relatively small, which limits the generalizability of the findings. Additionally, the retrospective nature of the study means that the data relied on medical records, which may have been incomplete or inconsistent. Given the variability in presentation and timing of chronic voiding dysfunction after midurethral sling placement, further prospective research is needed to define standardized care pathways. These should include objective criteria for diagnosis, timing of surgical removal or incision, and postoperative follow-up strategies, in order to optimize patient outcomes and reduce unnecessary delays in treatment.

## 5. Conclusions

This study highlights the significant improvement in urinary symptoms following sling removal in women with chronic voiding dysfunction after suburethral sling procedures for stress urinary incontinence. Despite a partial recurrence of SUI in some cases, these findings suggest that sling removal is an effective strategy for resolving urinary symptoms, emphasizing the importance of timely and individualized surgical intervention in managing complications associated with sling procedures.

## Figures and Tables

**Table 1:** Preoperative uroflowmetry and urodynamics. Continuous data is presented as mean ± standard deviation. Non-continuous data as absolute (relative) frequency. BCI = Bladder Contractility Index; pDet@Qmax = Detrusor Pressure at Maximum Flow; Qmax = Maximum Flow; MCC = Maximum Cystometric Capacity; PVR = PostVoid Residual; SUI = Urodynamic Stress Urinary Incontinence.

**Uroflowmetry**	
Maximum urine flow rate (mL/s)	12.9 ± 6.4
Flow time (s)	40.6 ± 21.3
PVR (mL)	153.1 ± 130
Positive PVR	7 (43.75%)
**Urodynamics**	
Detrusor hyperactivity	6 (37.5%)
SUI	0
pDet@Qmax (cmH_2_O)	25.1 ± 17.1
Qmax (mL/s)	12.2 ± 6.3
BCI	91.9 ± 14.4
MCC (mL)	364.8 ± 72.5
PVR (mL)	162.2 ± 14.4
Positive PVR	8 (50%)

**Table 2:** Preoperative and postoperative symptoms. Data as absolute (relative) frequency. UUI = urge urinary incontinence; SUI = stress urinary incontinence; LUTS = Lower Urinary Tract Symptoms.

	Preoperative Symptoms	Postoperative Symptoms	*p*-Value
Voiding difficulties	16 (100%)	1 (6.75%)	<0.001
Recurrent UTI	6 (37.5%)	1 (6.5%)	0.083
UUI	5 (31.25%)	3 (18.75%)	0.685
SUI	4 (25%)	6 (37.5%)	0.704
LUTS	4 (25%)	0	0.101
Urinary urgency	3 (18.75%)	0	0.226
Nocturia	1 (6.5%)	0	1
Dyspareunia	1 (6.5%)	0	1
Asymptomatic	0	7 (43.75%)	0.007

## Data Availability

No new data were created or analyzed in this study.

## Supplementary Materials

The following supporting information can be downloaded at: https://www.mdpi.com/article/10.3390/healthcare13131517/s1, STROBE Statement—Checklist of items that should be included in reports of cross-sectional studies.

## Abbreviations

The following abbreviations are used in this manuscript:

SUI	Stress Urinary Incontinence
SIS	Single-incision slings
BOO	Bladder outlet obstruction
TOT	Transobturator Tape
TVT	Tension-free Vaginal Tape
BCI	Bladder Contractility Index
pDet@Qmax	Detrusor Pressure at Maximum Flow
Qmax	Maximum Flow
MCC	Maximum Cystometric Capacity
PVR	PostVoid Residual
SUI	Urodynamic Stress Urinary Incontinence
UUI	Urge Urinary Incontinence
LUTS	Lower Urinary Tract Symptoms
